# Supplementary material for: A cellular automaton model of osteogenic differentiation reveals identifiability limits of endpoint assays
Source: Front Syst Biol. 2026 Aug 17;6:1872774. doi: 10.3389/fsysb.2026.1872774 (PMC13526594; doi:10.3389/fsysb.2026.1872774)
Supplement: Supplementary file 1 [file DataSheet1.pdf]

Supplementary Information

Contents

1 Full model specification **3**
1.1 Spatial and temporal representation . . . 3
1.2 Migration rule and calibration . . . 3
1.3 Osteogenic commitment . . . 4
1.4 Ascorbic acid dynamics . . . 5

2 Parameterization and calibration **7**
2.1 Basal and osteogenic marker kinetics . . . 7
2.2 Mineralization dynamics . . . 8
2.3 Dex and MK-4 compound–response relationships . . . 8
2.4 General parameter choices . . . 9
2.5 Seeding estimates . . . 10

3 Sensitivity and perturbation analyses **11**
3.1 Structural ablation and nonspatial comparator . . . 11
3.1.1 Nonspatial comparator . . . 11
3.2 Selected Dex/MK-4 dose ranges for analysis . . . 12
3.3 Perturbation-family identifiability analysis . . . 13
3.4 Sensitivity on alternative parameterizations . . . 13

4 Spatial and image-based analyses **13**
4.1 Spatial autocorrelation . . . 13
4.2 Nuclear morphology analysis . . . 17

5 Supplementary Figures **18**

6 Reagents Information **20**

1 Full model specification

This document provides detailed mathematical formulations, parameter estimation procedures, sensitivity analyses, and supplementary experimental figures supporting the OsteoMin model.

1.1 Spatial and temporal representation

The model is implemented on a two-dimensional square lattice of size $n \times n$ representing the culture surface. Each lattice site corresponds to a spatial area of

$$\Delta x = 30\,\mu\text{m},$$

approximately the diameter of an MC3T3-E1 cell consistent with microscopy observations in MC3T3 cultures [1].

1.2 Migration rule and calibration

Pre-OB migration was modeled as a stochastic random walk on a two-dimensional lattice. At each timestep ($\Delta t = 1\,\text{h}$), a cell attempts to move to a randomly selected empty Moore-neighbor site with probability p_m.

To reduce lattice anisotropy, the four axial directions were assigned a total probability weight of 2/3 and the four diagonal directions a total weight of 1/3. Thus, the probability of selecting a specific axial direction is $p_m/6$, while that of selecting a specific diagonal direction is $p_m/12$. For lattice spacing a, axial moves have squared length a^2 and diagonal moves have squared length $2a^2$, giving an expected squared displacement per timestep

$$\langle \Delta r^2 \rangle = 4\left(\frac{p_m}{6}\right)a^2 + 4\left(\frac{p_m}{12}\right)(2a^2) = \frac{4}{3}p_m a^2.$$

Since $\Delta t = 1\,\text{h}$, the model predicts

$$\langle r^2(t) \rangle = \frac{4}{3}p_m a^2\,t.$$

The migration probability p_m was calibrated to reproduce the migration of MC3T3-E1 cells on flat substrates reported in [1]. In Fig. 5b of that study, the flat condition reaches an RMS displacement of approximately $70\,\mu$m after ~ 16 h. This corresponds to a mean-squared displacement of approximately

$$\langle r^2 \rangle \approx (70\,\mu\text{m})^2 = 4900\,\mu\text{m}^2.$$

Assuming approximately diffusive scaling at long times gives an effective displacement rate

$$\frac{\langle r^2 \rangle}{t} \approx \frac{4900}{16} \approx 320\,\mu\text{m}^2/\text{h}.$$

Matching this experimental scaling to the lattice model yields

$$\frac{4}{3} p_m a^2 = 320\,\mu\text{m}^2/\text{h}.$$

Using the simulation lattice spacing $a = 30\,\mu$m gives

$$p_m = \frac{320}{(4/3) \times 30^2} \approx 0.27.$$

This value was used in all simulations.

1.3 Osteogenic commitment

Pre-OB differentiation was implemented as a delayed stochastic transition. Osteogenic induction in the simulations was initiated at $t_{\text{osteo}} = 3$ days (72 h), corresponding to the experimental time point at which osteogenic conditions were applied.

Cells remain in the pre-OB state for a maturation interval t_d after induction. After this delay, commitment occurs with a constant rate k_{commit}. The cumulative probability that a cell has committed by time t is

$$f_{OB}(t) = \begin{cases} 0, & t < t_{\text{osteo}} + t_d, \\ 1 - e^{-k_{\text{commit}}(t - t_{\text{osteo}} - t_d)}, & t \geq t_{\text{osteo}} + t_d. \end{cases}$$

The delay parameter reflects the experimentally observed lag between osteogenic induction and early differentiation markers in MC3T3-E1 cultures. Transcriptomic analysis indicates that the transition from proliferation to osteogenic programs occurs around day 2–4 following induction [2]. We therefore set the maturation delay to $t_d = 3$ days (72 h), consistent with our experimental protocol in which osteogenic supplementation is introduced at day 3 after seeding.

Following this delay, commitment occurs with constant rate $k_{\text{commit}} = 0.0125\,\text{h}^{-1}$, corresponding to $0.30\,\text{day}^{-1}$. This implies a characteristic commitment timescale of approximately

10 days after the maturation delay:

$$T_{95} = \frac{-\ln(0.05)}{k_{\text{commit}}} \approx 10 \text{ days.}$$

Thus, most cells transition to the OB state approximately 10 days after the delay period, consistent with reported timing of MC3T3-E1 differentiation and mineralization onset between days 14–21 following osteogenic induction [2, 3].

The resulting temporal evolution of pre-OB and OB population counts under baseline osteogenic conditions is shown in Fig. 1.

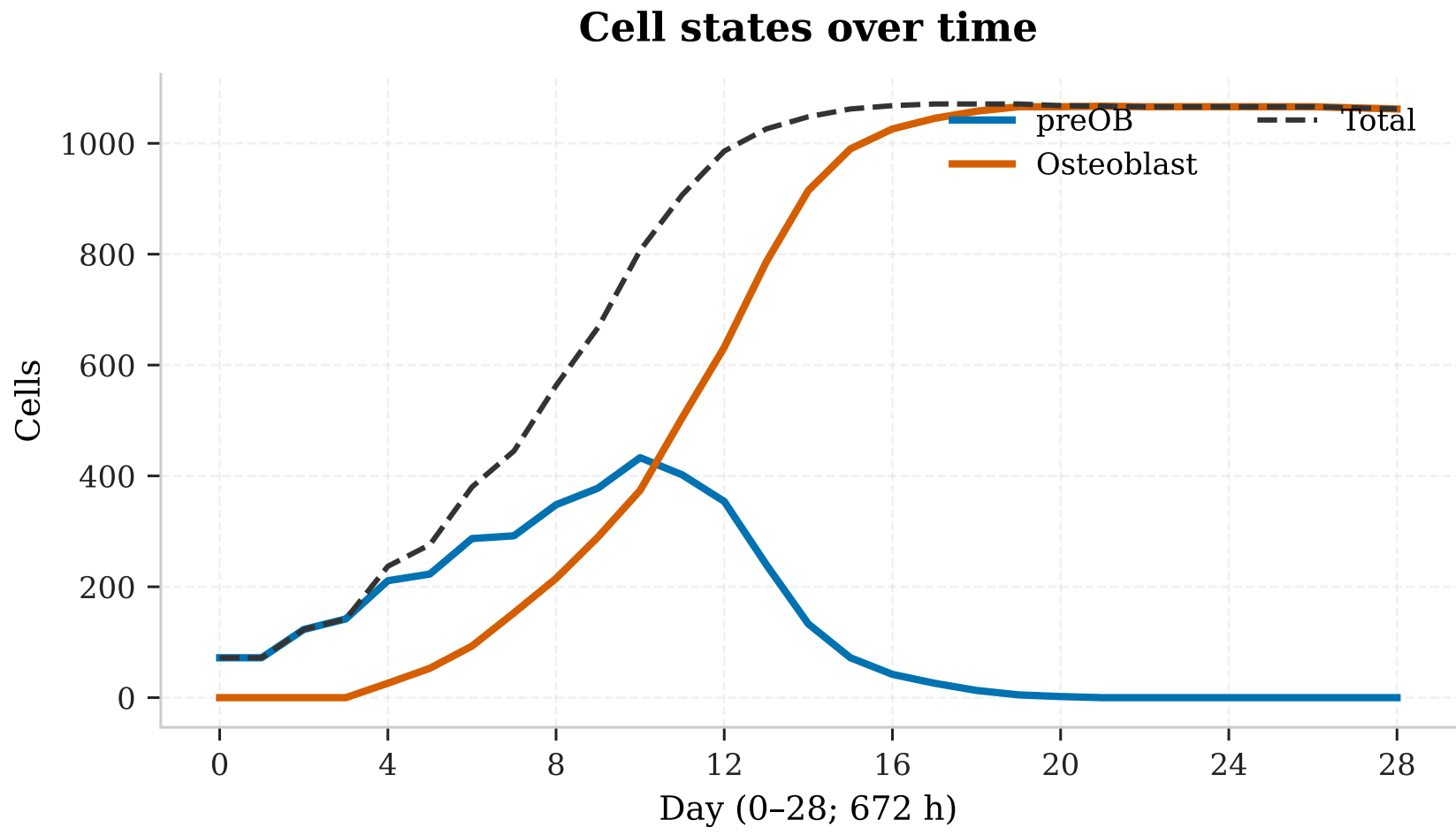

Figure 1: Temporal progression of pre-OB and OB population counts in the spatial OsteoMin model under baseline osteogenic conditions.

1.4 Ascorbic acid dynamics

Ascorbic acid (AA) is supplied with osteogenic medium exchange and undergoes first-order oxidative degradation between exchanges.

The decay constant is

$$k_{AA} = \frac{\ln 2}{t_{1/2}^{AA}}, \qquad t_{1/2}^{AA} \approx 8 \text{ h.}$$

Medium is replaced every m days, corresponding to an exchange interval

$$\tau_m = 24m \text{ h.}$$

AA supplementation begins simultaneously with osteogenic induction at t_{osteo}.

Define the medium-refresh indicator

$$u_{\text{med}}(t_k) = \begin{cases} 1, & u_{\text{osteo}}(t_k) = 1 \text{ and } \operatorname{mod}(t_k - t_{\text{osteo}}, \tau_m) = 0, \\ 0, & \text{otherwise.} \end{cases}$$

Between medium exchanges, AA decays exponentially:

$$c_{AA}(t) = C_{AA,0} \exp(-k_{AA}(t - t_p)), \qquad t \geq t_p, \tag{1}$$

where t_p denotes the most recent medium exchange time.

The discrete-time update consistent with timestep $\Delta t = 1$ h is

$$c_{AA}(t_{k+1}) = c_{AA}(t_k) e^{-k_{AA}\Delta t} + C_{AA,0}\, u_{\text{med}}(t_k). \tag{2}$$

Initial condition:

$$c_{AA}(t_k) = 0, \qquad t_k < t_{\text{osteo}}.$$

Here $C_{AA,0}$ denotes the AA concentration supplied at each medium exchange. The resulting trajectory represents repeated AA pulses followed by exponential decay across successive exchange cycles. The corresponding time course of effective ascorbic acid availability under repeated medium exchange is shown in Fig. 2.

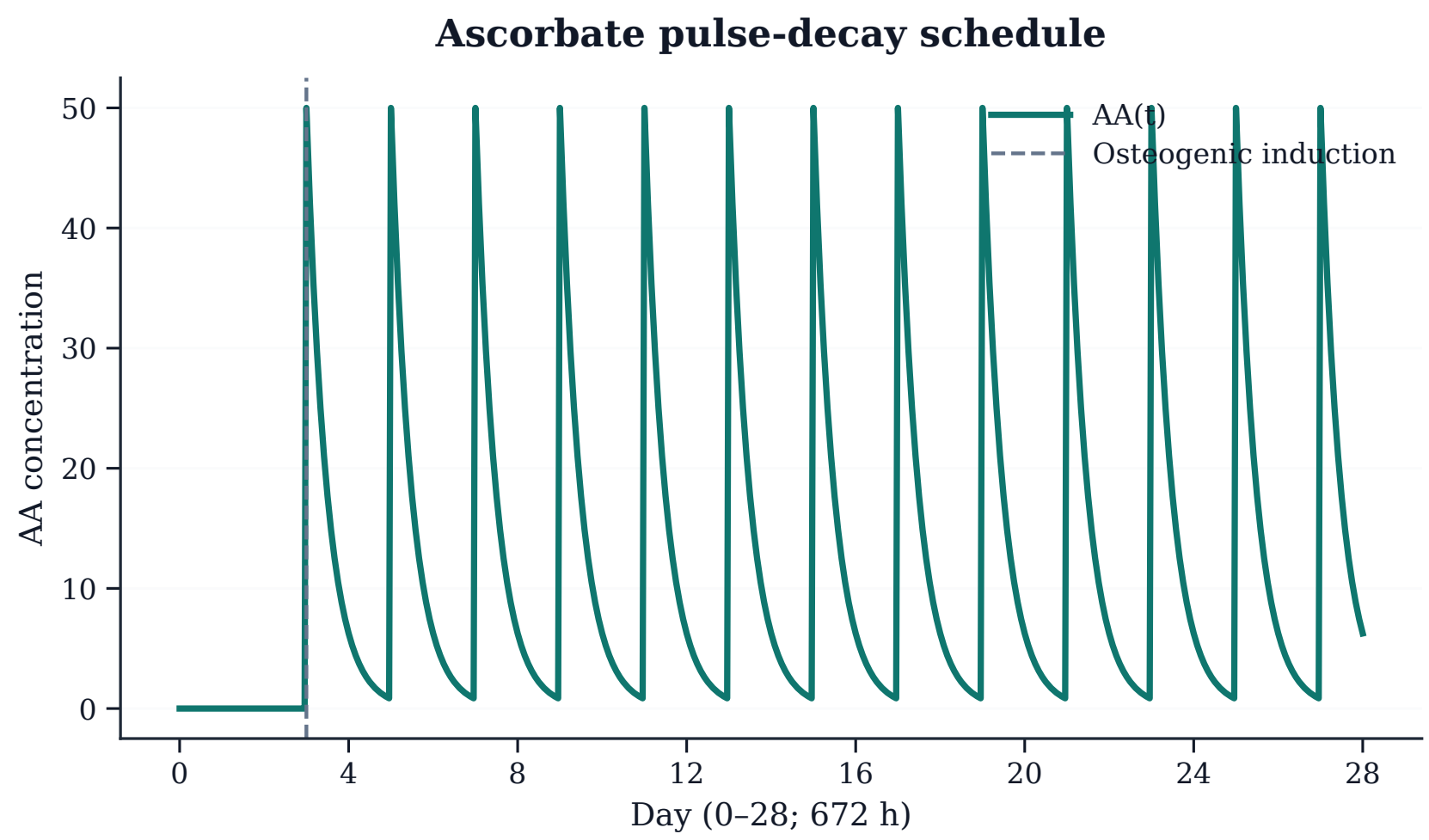

Figure 2: Effective ascorbic acid concentration in the medium under repeated supplementation and exponential degradation.

2 Parameterization and calibration

Published time-course and dose–response datasets were used to estimate effective phenomenological parameters describing marker production and compound modulation. Functional forms were selected to reproduce dominant experimental trends while limiting the number of poorly identifiable parameters.

2.1 Basal and osteogenic marker kinetics

Early osteogenic markers such as alkaline phosphatase (ALP) activity and collagen deposition exhibit approximately monotonic increases over the experimentally observed time windows. These dynamics were therefore approximated using constant-rate accumulation models:

$$M(t) = M_0 + rt \tag{3}$$

where $M(t)$ denotes marker signal, M_0 the baseline level, and r an effective production rate. Separate basal (r_b) and osteogenic (r_o) contributions were estimated from literature datasets.

ALP production rates were estimated from measurements at days 3, 7, 14, and 21 reported in [4]. Least-squares fitting yielded

$$r_{b,A} = 9.49 \times 10^{-2}\,\mathrm{AU\,h^{-1}}, \qquad r_{o,A} = 8.10 \times 10^{-1}\,\mathrm{AU\,h^{-1}},$$

with fit errors RMSE = 5.52 AU (basal) and 32.1 AU (osteogenic), indicating that the linear approximation captures the overall temporal increase in ALP activity over the observed interval.

Collagen production rates were estimated from measurements obtained under basal and ascorbic-acid-supplemented conditions at days 1–12 [5]. Least-squares fitting yielded

$$r_{b,C} = 3.36 \times 10^{-3}\,\mathrm{AU\,h^{-1}}, \qquad r_{o,C} = 1.30 \times 10^{-2}\,\mathrm{AU\,h^{-1}},$$

with RMSE $= 9.14 \times 10^{-2}$ AU (basal) and 3.79×10^{-1} AU (osteogenic). The approximately linear increase in collagen signal across the measured time window supports the constant-rate approximation.

2.2 Mineralization dynamics

Mineral accumulation exhibits delayed onset followed by nonlinear increase reflecting extracellular matrix maturation and hydroxyapatite nucleation. Alizarin Red S (ARS) time-course measurements reported in [6] were approximated using a Hill-type activation model:

$$H(t) = H_0 + r_{o,H} \frac{t^{n_H}}{\tau_H^{n_H} + t^{n_H}} \tag{4}$$

where τ_H defines the characteristic activation timescale and n_H controls transition steepness. Fitting to measurements at days 4, 7, 10, 14, 21, and 28 yielded

$$r_{o,H} = 1.03 \times 10^{-1}\,\mathrm{AU\,h^{-1}}, \qquad \tau_H = 384\,\mathrm{h}, \qquad n_H = 2,$$

with RMSE = 2.25 AU. The Hill formulation reproduces the delayed onset and subsequent nonlinear increase in ARS signal commonly observed during MC3T3-E1 differentiation.

2.3 Dex and MK-4 compound–response relationships

Dose–response effects of dexamethasone (Dex) and vitamin K_2 (MK-4) were estimated from published single-agent datasets using nonlinear least-squares regression. Experimental studies frequently report biphasic responses to osteogenic supplements, with intermediate concentrations producing maximal marker expression.

Dose-dependent modulation was therefore approximated using a log-parabolic response function applied multiplicatively to baseline marker production:

$$y(c) = y_0 \left[1 + a \left(1 - b \left(\log_{10} \frac{c}{c_0}\right)^2\right)\right] \tag{5}$$

where c denotes compound concentration, c_0 the concentration associated with maximal response, a the response amplitude, and b controls response width in log-dose space.

Dex-dependent ALP activity was fitted to measurements at 0–100 nM [7], yielding

$$c_{D,0,A} = 22.76\,\mathrm{nM}, \quad a_{D,A} = 3.72, \quad b_{D,A} = 1.75.$$

Dex-dependent collagen response was fitted to measurements at 0–10 nM [8], yielding

$$c_{D,0,C} = 17.27\,\mathrm{nM}, \quad a_{D,C} = 2.65, \quad b_{D,C} = 0.814.$$

Dex-dependent mineralization was fitted to measurements at 0–100 nM [9], yielding

$$c_{D,0,H} = 67.79\,\text{nM}, \quad a_{D,H} = 1.15, \quad b_{D,H} = 0.345.$$

MK-4-dependent ALP activity was fitted to measurements at 0–10 μM [10], yielding

$$c_{K,0,A} = 0.7829\,\mu\text{M}, \quad a_{K,A} = 1.095, \quad b_{K,A} = 1.058.$$

Quantitative MK-4 collagen dose–response data were available only for control and 10 μM [11]. Because these measurements do not independently constrain response curvature, shape parameters were fixed to mineralization-derived values ($a_{K,C} = 4.663$, $b_{K,C} = 2.145$), yielding

$$c_{K,0,C} = 0.3480\,\mu\text{M}.$$

MK-4-dependent mineralization was fitted to measurements at 0–10 μM [10], yielding

$$c_{K,0,H} = 0.6692\,\mu\text{M}, \quad a_{K,H} = 4.665, \quad b_{K,H} = 2.150.$$

For sparse dose–response datasets ($n \leq p$), fitted coefficients should be interpreted as effective phenomenological calibration parameters rather than uniquely identifiable mechanistic constants.

Table 1: **Literature-derived and fitted production/modulation parameters.**

**Fit**	n	DoF	RMSE	**Estimated parameters**	**Source**
Dex–ALP prod.	4	0	5.43×10^{-12}	$c_{D,0,A} = 22.76\,\text{nM},\ a_{D,A} = 3.72,\ b_{D,A} = 1.75$	[7]
Dex–coll. prod.	3	NA	3.02×10^{-9}	$c_{D,0,C} = 17.27\,\text{nM},\ a_{D,C} = 2.65,\ b_{D,C} = 0.814$	[8]
Dex–mineral dep.	3	NA	5.13×10^{-9}	$c_{D,0,H} = 67.79\,\text{nM},\ a_{D,H} = 1.15,\ b_{D,H} = 0.345$	[9]
MK-4–ALP prod.	4	0	2.04×10^{-15}	$c_{K,0,A} = 0.7829\,\mu\text{M},\ a_{K,A} = 1.095,\ b_{K,A} = 1.058$	[10]
MK-4–coll. prod.	2	0	7.85×10^{-15}	$c_{K,0,C} = 0.3480\,\mu\text{M},\ a_{K,C} = 4.663,\ b_{K,C} = 2.145$	[11]
MK-4–mineral dep.	4	0	2.84×10^{-14}	$c_{K,0,H} = 0.6692\,\mu\text{M},\ a_{K,H} = 4.665,\ b_{K,H} = 2.150$	[10]

Compound–response parameters were weakly constrained because only limited literature measurements were available and should therefore be interpreted as effective phenomenological coefficients rather than uniquely estimated biological constants.

2.4 General parameter choices

Ascorbic acid (AA) availability was modeled as an exponentially decaying signal reflecting its rapid degradation in cell culture medium. The initial concentration was set to 50 μg/mL corre-

sponds to 0.284 mM AA, consistent with standard osteogenic culture conditions, and decayed with an effective half-life of 8 h, consistent with reported ascorbate instability in culture media [12]. Collagen production was modulated by AA availability using a saturating gate with half-saturation constant $K_{A,C} = 5\,\mu\text{g/mL}$.

Delayed collagen support for mineralization is controlled by the maturation timescale τ_{col}. Experimental studies show that extracellular matrix deposition precedes mineral formation and that hydroxyapatite nucleation requires a sufficiently developed collagen scaffold [5, 13]. We therefore set $\tau_{\text{col}} = 48$ h as an effective matrix maturation timescale.

Because collagen provides nucleation sites for hydroxyapatite formation, mineral deposition depends on extracellular matrix availability [13]. This dependence is represented phenomenologically using a saturating function with half-saturation constant $K_{\text{col}} = 1.0$ in normalized collagen units, defining the matrix level at which mineral deposition reaches half-maximal rate.

The parameters τ_H and n_H determine the characteristic timescale and steepness of mineralization activation following OB commitment. To reproduce the delayed onset and progressive increase in Alizarin Red S staining observed during MC3T3-E1 differentiation, we fixed $\tau_H = 384\,\text{h}$ and $n_H = 2$. The osteogenic mineral deposition rate was set to $r_{o,H} = 0.103\,\text{AU}\,\text{h}^{-1}$, obtained from fitting mineral accumulation dynamics reported in [6].

2.5 Seeding estimates

Cell concentration was determined experimentally by hemocytometer counting following a 10-fold dilution. A total of 49 cells were counted across 12 large squares, corresponding to an average of $49/12 \approx 4.1$ cells per square. Since each large square represents 10^{-4} mL, the diluted suspension concentration was 4.1×10^4 cells/mL. After correcting for dilution, the original cell concentration was estimated as 4.1×10^5 cells/mL.

Each well received $20\,\mu\text{L}$ of the cell suspension, corresponding to approximately 8.0×10^3 cells per well.

Cells were seeded onto 12 mm diameter glass coverslips with a surface area $A = \pi r^2 \approx 1.13\,\text{cm}^2$. This yields an initial seeding density of

$$\rho \approx 7.1 \times 10^3 \text{ cells/cm}^2 \quad (\approx 72 \text{ cells/mm}^2).$$

3 Sensitivity and perturbation analyses

All parameters were sampled independently from uniform distributions using Saltelli sampling.

Table 2: Parameter ranges used for Sobol sensitivity analysis.

Parameter	Description	Lower	Upper
ALP basal prod.	ALP basal production rate	0.07	0.13
ALP osteogenic prod.	ALP osteogenic production rate	0.60	1.05
Collagen basal prod.	Collagen basal production rate	0.0020	0.0055
Collagen osteogenic prod.	Collagen osteogenic production rate	0.0080	0.0200
Mineral osteogenic dep.	Mineral osteogenic deposition rate	0.075	0.150
Mineral Hill n	Mineral Hill function n	1.5	3.5
Mineralization tau	Mineralization activation timescale	288.0	480.0
Collagen maturation tau	Collagen maturation timescale (τ_{col})	24.0	72.0
Collagen support K	Collagen support half-saturation (K_{col})	0.5	2.0
Commitment rate	Commitment rate	0.0075	0.0200
Pre-OB apoptosis prob.	Pre-OB apoptosis probability	0.0005	0.0015
Cell motility prob.	Cell motility probability	0.15	0.40
Ascorbate half-life	Ascorbate half-life	4.0	12.0
Collagen AA K	AA half-saturation for collagen ($K_{A,C}$)	2.5	8.0
Commitment delay	Commitment delay	24.0	72.0
Min division interval	Minimum division interval	30.0	46.0
Division period	Division period	8.0	24.0
Division prob.	Division attempt probability	0.75	1.0
Osteogenic induction	Osteogenic induction time	48.0	96.0
Media refresh interval	Media refresh interval	24.0	72.0

3.1 Structural ablation and nonspatial comparator

We assessed the contribution of key mechanisms in the spatial osteogenesis model by systematically removing individual processes and comparing results with a matched nonspatial (well-mixed) formulation. Analyses correspond to condition C2 (Dex = 0, MK-4 = 0).

The reference system was the full spatial model initialized from the baseline configuration. Simulations were performed for 28 days using 10 replicate seeds. Endpoints were ALP (day 10), collagen (day 14), mineralization (day 28), and total cell number (day 28).

3.1.1 Nonspatial comparator

To isolate the contribution of spatial organization, we constructed a nonspatial population-level formulation in which the explicit lattice representation of the agent-based model (ABM) is removed while preserving the same kinetic parameterization and assay mappings. Initial

conditions were matched by setting the starting pre-OB and OB population counts equal to the day-0 reference state of the spatial simulation. The effective population capacity was fixed to $K = 1089$, corresponding to the number of lattice sites in the spatial model (33×33 grid representing $1\,\mathrm{mm} \times 1\,\mathrm{mm}$). Model outputs were evaluated at the same assay-aligned time points as the spatial simulations (day 10 ALP, day 14 collagen, day 28 mineral, and day-28 total cell number). Because the nonspatial formulation does not include stochastic cell-level events, replicate variability was introduced by applying log-normal perturbations (coefficient of variation 0.10) to selected kinetic rate parameters.

In the nonspatial formulation, cells are not assigned spatial positions. Instead, the numbers of pre-OB and OB cells are represented as total population counts that evolve in time according to the same kinetic rate parameters used in the spatial model. Cell proliferation is limited by total population size relative to the carrying capacity K, replacing the spatial occupancy constraint that restricts daughter-cell placement in the ABM lattice. Differentiation commitment from pre-OB to OB is implemented as a rate-dependent population transfer after the same induction delay used in the spatial model, but without stochastic per-cell transitions. As in the spatial model, cell division contributes newly generated cells to the pre-OB population regardless of parent state, preserving the same lineage structure.

Extracellular matrix production and maturation follow identical kinetic expressions as in the spatial model but are evaluated using population-averaged quantities rather than spatially resolved fields. Consequently, mineral formation is determined by the global availability of mature collagen and the average progression of OB cells beyond commitment, rather than requiring spatial co-localization of osteoblast occupancy and sufficiently matured matrix at individual lattice sites. This formulation preserves the same biochemical rate structure and assay observables as the spatial ABM while removing spatial constraints on cell placement, local crowding effects, and stochastic variation in commitment timing. Differences between the two formulations therefore reflect the contribution of spatial exclusion, spatially heterogeneous matrix accumulation, and asynchronous single-cell state transitions to emergent mineralization dynamics.

3.2 Selected Dex/MK-4 dose ranges for analysis

Dose selections were defined for single-compound variations and combined Dex–MK-4 response mapping.

Table 3: **Single-compound dose ranges.** One compound was varied while the other was fixed at zero. The baseline condition corresponds to Dex = 0 nM and MK-4 = 0 μM.

**Condition**	**Dose values**
MK-4 only	Dex = 0 nM; 0.03, 0.10, 0.20, 0.30, 0.60, 1.00, 2.50, 5.00, 10 μM
Dex only	MK-4 = 0 μM; 0.3, 1, 2, 3, 6, 10, 20, 30, 60, 100 nM
Baseline	Dex = 0 nM; MK-4 = 0 μM

Table 4: **Combined Dex–MK-4 dose ranges.** All pairwise combinations of the listed Dex and MK-4 concentrations were evaluated, yielding 100 total conditions.

**Compound**	**Dose values**
Dex	0, 1, 5, 10, 20, 30, 40, 60, 80, 100 nM
MK-4	0, 0.1, 0.3, 0.5, 1, 2, 3, 5, 7, 10 μM

3.3 Perturbation-family identifiability analysis

To assess whether assay-aligned endpoints distinguish mechanistic perturbations, parameters were varied within biologically plausible ranges using family-wise quasi-random sampling. For each perturbation family, only the associated parameters were varied, while all others were fixed at baseline values. Sampling schemes were adapted to parameter type and structural constraints.

3.4 Sensitivity on alternative parameterizations

The sensitivity of the trajectories-based approach proposed here was assessed by computing the family confusion matrices and the macro-F1 score while allowing the main parameters to vary in their reported ranges using Saltelli's quasi-random sampling (see Table 6 and Fig. 3).

4 Spatial and image-based analyses

4.1 Spatial autocorrelation

Let $H(x, y)$ denote the final mineral field on our grid. The mean-subtracted field is

$$\tilde{H}(x, y) = H(x, y) - \bar{H}, \qquad \bar{H} = \frac{1}{WH} \sum_{x,y} H(x, y). \tag{6}$$

The unnormalized autocovariance was computed using the Wiener–Khinchin theorem

$$C_{\text{raw}}(\Delta x, \Delta y) = \text{IFFT2}\left(\left| \text{FFT2}(\tilde{H}) \right|^2 \right), \tag{7}$$

Table 5: Parameter ranges used for perturbation-family identifiability analysis. Each family was varied independently around the baseline parameter set.

Parameter	Description	Lower	Upper
**Spatial pattern**			
init_slug	Initial spatial seeding motif	C01	C50
boundary_scale	Spatial scaling factor	fixed	fixed
**Cell number**			
init_cells	Initial number of pre-OB cells	27	212
grid_spacing	Structured seeding grid spacing	1	4
**Cell dynamics**			
osteo_induction_hours	Osteogenic induction time (h)	50	95
commit_delay	Commitment delay t_d (h)	24	71
commit_k	Commitment rate k_{commit}	0.00825	0.01970
move_prob	Cell motility probability p_m	0.1536	0.3929
min_div_hours	Minimum division interval (h)	32	45
max_div_hours	Maximum division interval (h)	43	62
apoptosis_prob	Pre-OB apoptosis probability	0.000547	0.001451
**Ascorbic acid / media chemistry**			
aa0	Initial AA concentration $C_{AA,0}$ (µg/mL)	25.72	89.13
aa_half_life	Ascorbate half-life $t_{1/2}^{AA}$ (h)	4.07	11.94
collagen_aa_km	AA half-saturation for collagen $K_{A,C}$	2.58	7.92
media_interval	Media refresh interval τ_m (h)	24	72

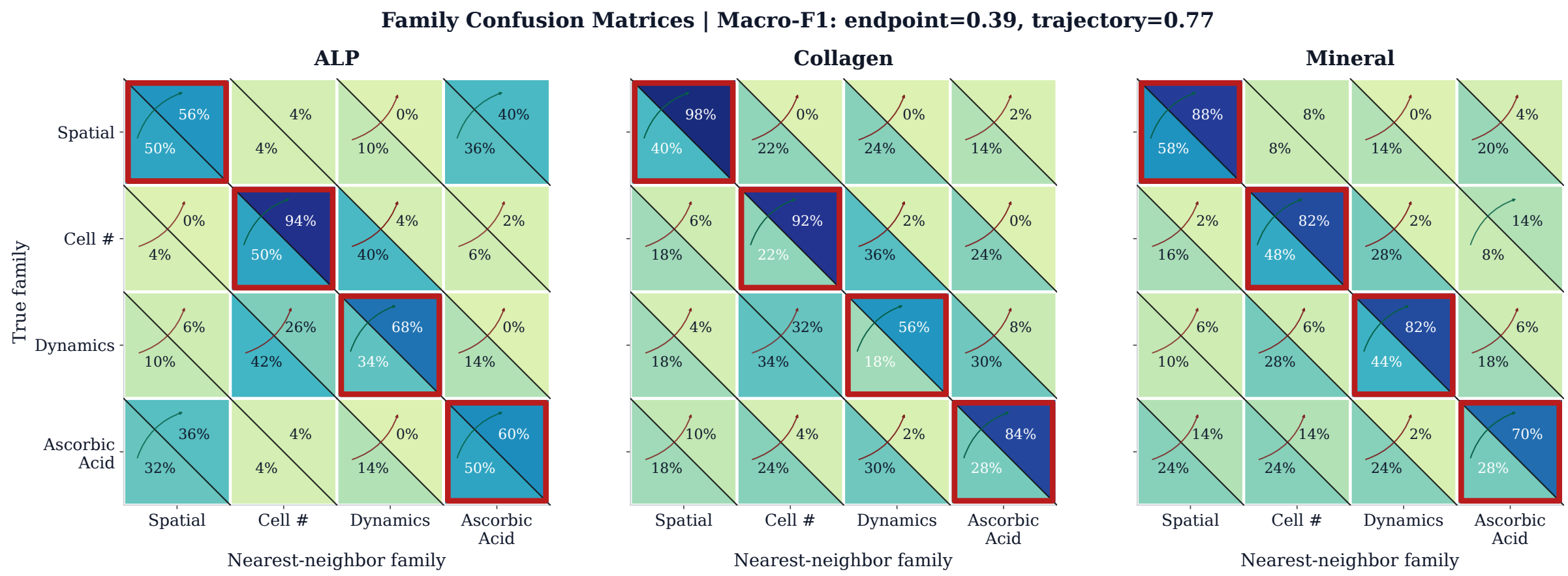

Figure 3: Classification of perturbation families from marker-specific readouts using split-cell confusion matrices for the sensitivity analysis using the parameter ranges of Table 6. Lower-left triangles show endpoint-based classification and upper-right triangles show trajectory-based classification over the corresponding. Red-outline squares denote true positives.

Table 6: Parameter ranges used for the sensitivity check on alternative parameterizations

Parameter	Description	Lower	Upper
osteo_induction_hours	Osteogenic induction time (h)	48	96
commit_delay	Commitment delay t_d (h)	24	72
commit_k	Commitment rate k_{commit}	0.0075	0.02
move_prob	Cell motility probability p_m	0.15	0.4
min_div_hours	Minimum division interval (h)	30	46
div_gap_hours	Division period (h)	8	24
apoptosis_prob	Pre-OB apoptosis probability	0.0005	0.0015
alp-rb	ALP basal production rate	0.07	0.13
alp-ro	ALP osteogenic production rate	0.6	1.05
collagen-rb	Collagen basal production rate	0.002	0.0055
collagen-ro	Collagen osteogenic production rate	0.008	0.02

and normalized as

$$C(\Delta x, \Delta y) = \frac{C_{\text{raw}}(\Delta x, \Delta y)}{C_{\text{raw}}(0,0)}. \tag{8}$$

Radial averaging was performed using periodic distances

$$d_x = \min(\Delta x,\, W - \Delta x), \qquad d_y = \min(\Delta y,\, H - \Delta y), \tag{9}$$

$$r = \sqrt{d_x^2 + d_y^2}, \tag{10}$$

with integer radial bins $i \in [0, \lfloor \min(W, H)/2 \rfloor)$. The radial correlation profile $C_r(i)$ was obtained by averaging $C(\Delta x, \Delta y)$ within each annulus.

The autocorrelation length ξ was defined as the first radius where $C_r(i)$ crossed e^{-1}, computed by linear interpolation between adjacent bins:

$$C_r(i-1) \geq e^{-1}, \qquad C_r(i) \leq e^{-1}. \tag{11}$$

For each condition, the reported value corresponds to the replicate mean

$$\xi = \mu_\xi \pm \sigma_\xi, \qquad \mu_\xi = \frac{1}{n} \sum_{m=1}^{n} \xi_m. \tag{12}$$

Distances are reported in lattice units.

Spatial heterogeneity of mineral deposition quantified from ARS images is summarized in Fig. 4.

Spatial organization was characterized using a sliding-window local variance metric (64×64

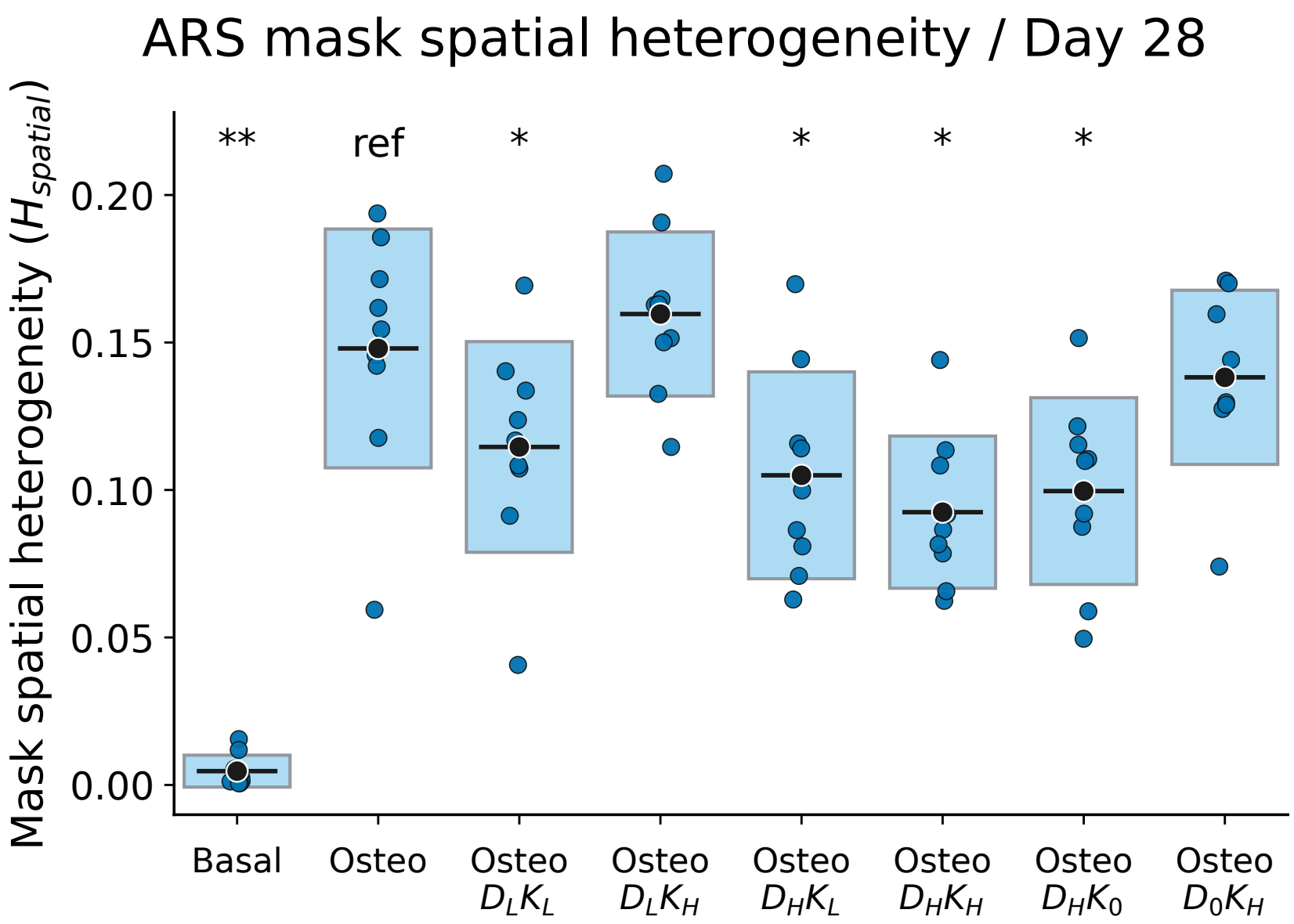

Figure 4: Spatial heterogeneity of mineral deposition at day 28 quantified from ARS images.

px), where heterogeneity (H_{spatial}) was defined as the image-averaged local variance. Replicate values ($n = 9$ per condition) are reported as mean ± SD. Statistical differences relative to the osteogenic reference condition (C2) were evaluated using two-sided Mann–Whitney U tests with Benjamini–Hochberg correction following an overall Kruskal–Wallis test. Representative spatial distributions of the simulated mineral field at day 28 across conditions are shown in Fig. 5.

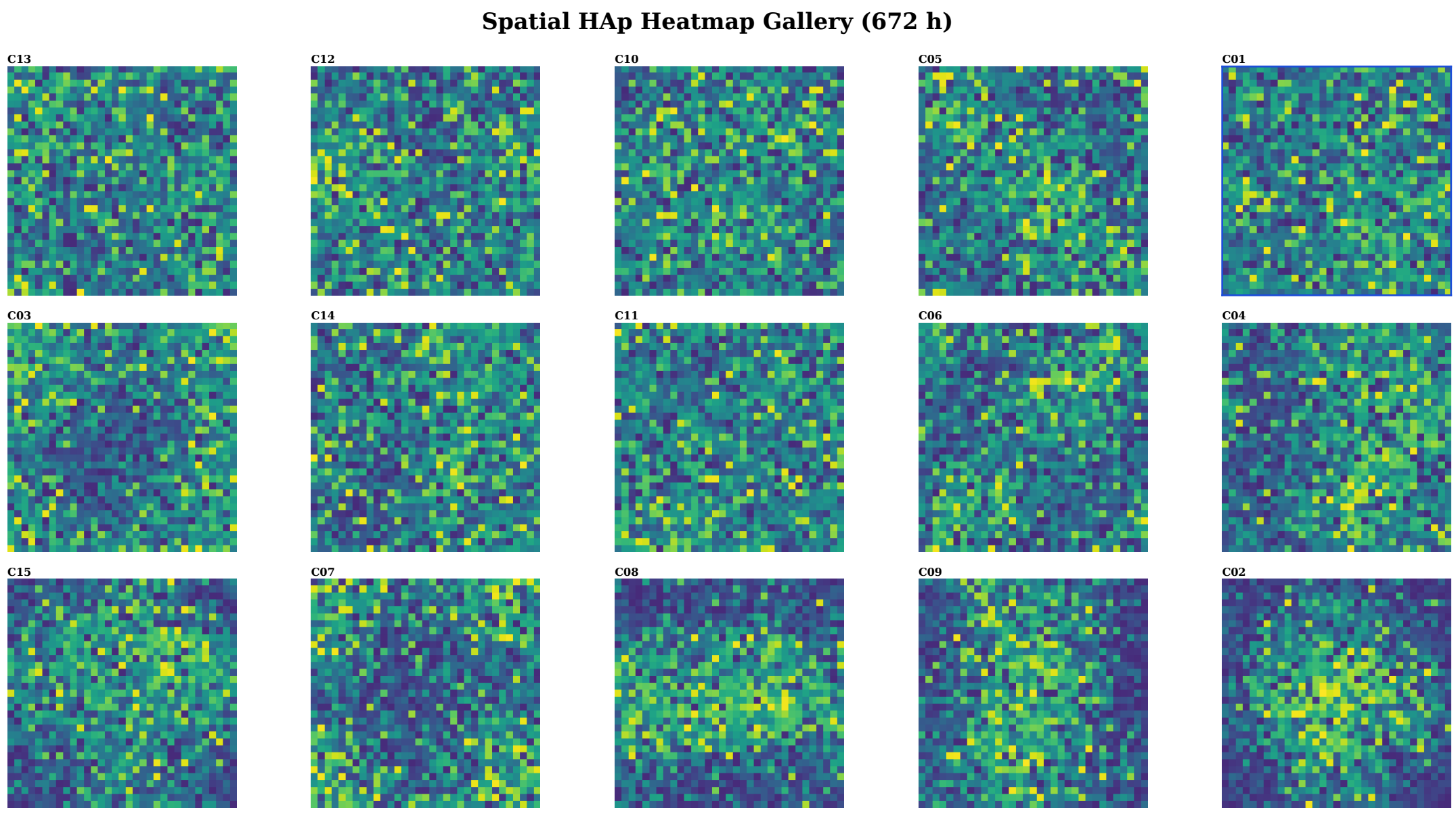

Figure 5: Spatial distributions of the simulated mineral field at day 28 across conditions.

High Dex conditions showed reduced spatial heterogeneity, indicating more spatially uniform mineral distribution, consistent with trends observed in nuclear morphology (DAPI area) from

confocal measurements.

4.2 Nuclear morphology analysis

DAPI-stained images were segmented by intensity thresholding to quantify nuclear projected area and aspect ratio, summarized in Figs. 6 and 7, respectively.

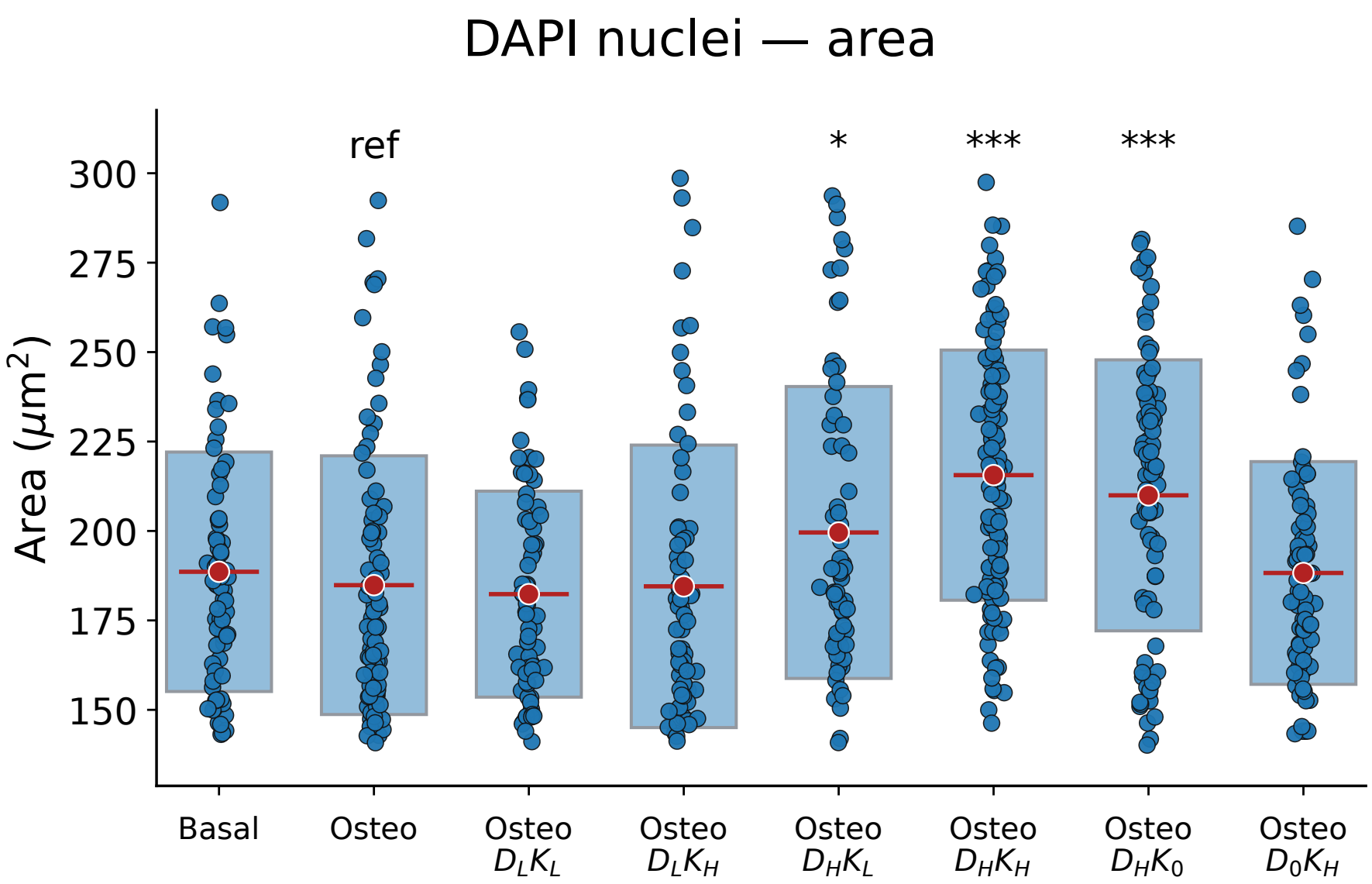

Figure 6: Nuclear projected area measured from DAPI-stained images across culture conditions.

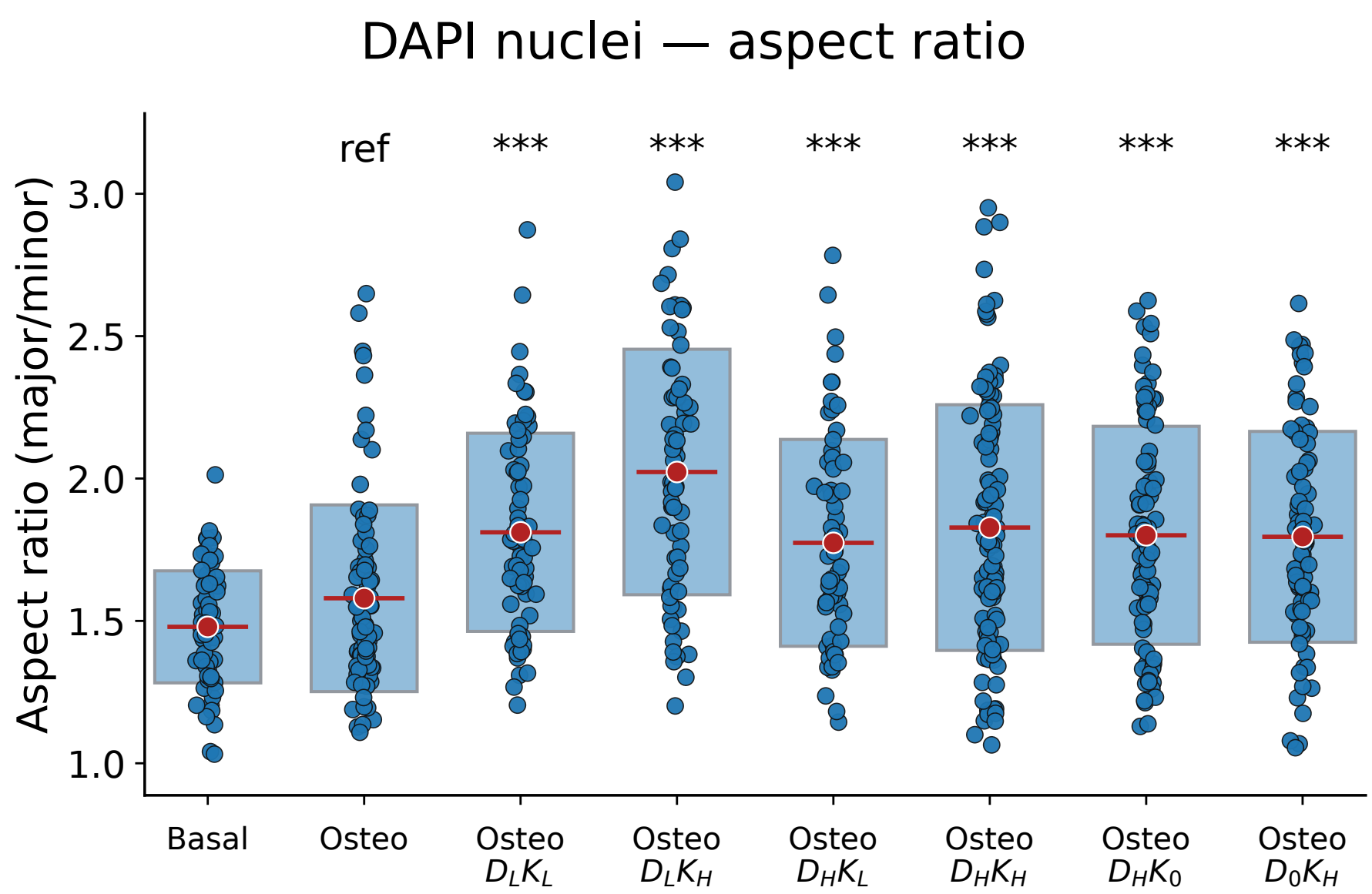

Figure 7: Nuclear aspect ratio (major/minor axis) measured from DAPI-stained images across culture conditions.

Nuclear morphology was analyzed from DAPI-stained images to assess condition-dependent changes in cell organization during osteogenic progression. Increases in nuclear projected area

and aspect ratio indicate cytoskeletal remodeling and more elongated cell states associated with differentiation and matrix production.

5 Supplementary Figures

Representative brightfield images across all experimental conditions are shown in Fig. 8. Differences in texture and local density reflect condition-dependent changes in proliferation, morphology, and matrix formation during osteogenic progression.

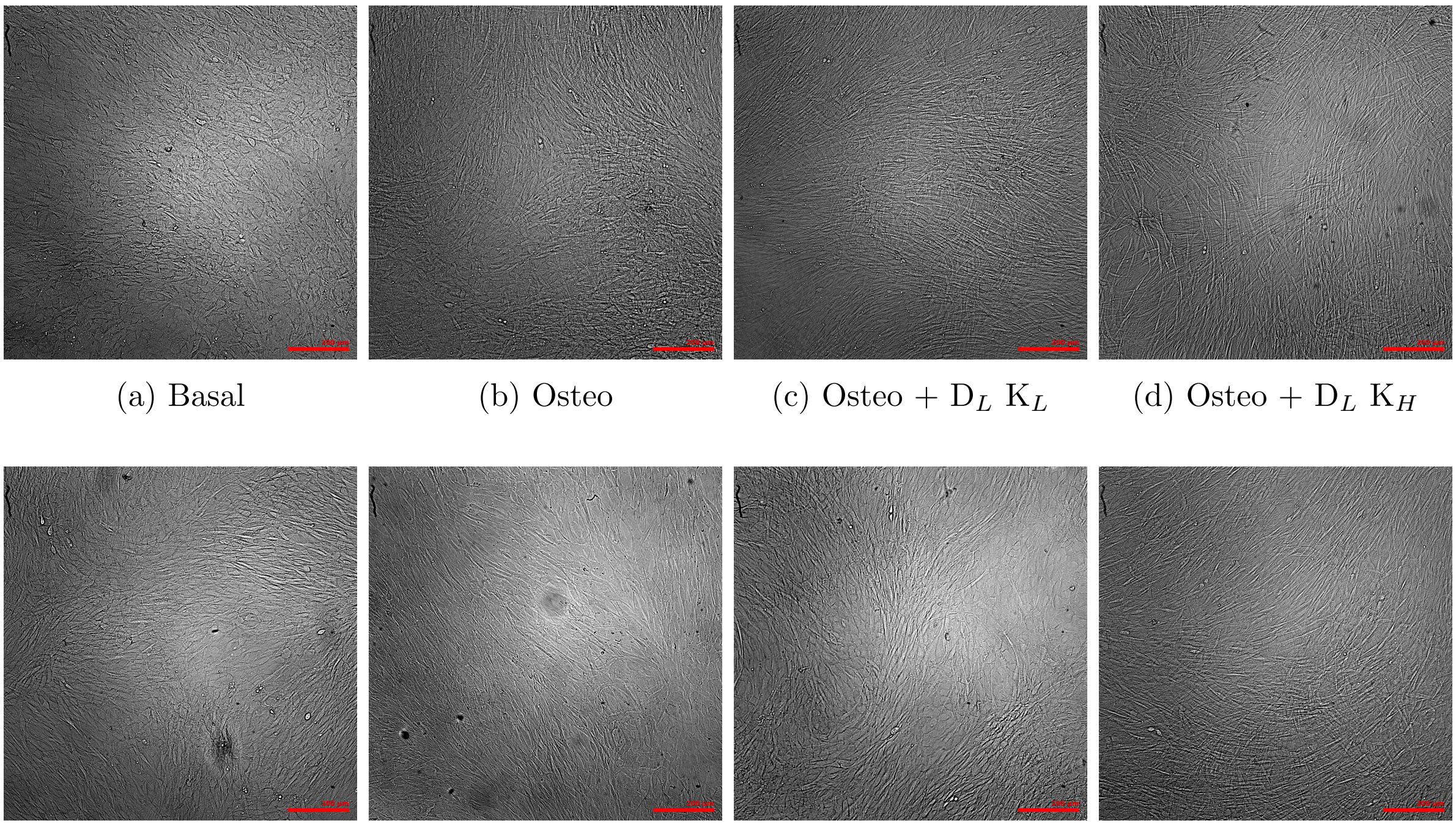

(a) Basal (b) Osteo (c) Osteo + D_L K_L (d) Osteo + D_L K_H

(e) Osteo + D_H K_L (f) Osteo + D_H K_H (g) Osteo + D_H K_0 (h) Osteo + D_0 K_H

Figure 8: Representative brightfield images acquired during culture across experimental conditions C1–C8.

Representative Alizarin Red S (ARS) images at day 28 across all experimental conditions are shown in Fig. 9. Spatial patterns indicate heterogeneous mineral deposition with localized nodular regions and condition-dependent differences in coverage and intensity.

The sensitivity of ARS-positive area quantification to the selected threshold is shown for a representative C2 image in Fig. 10.

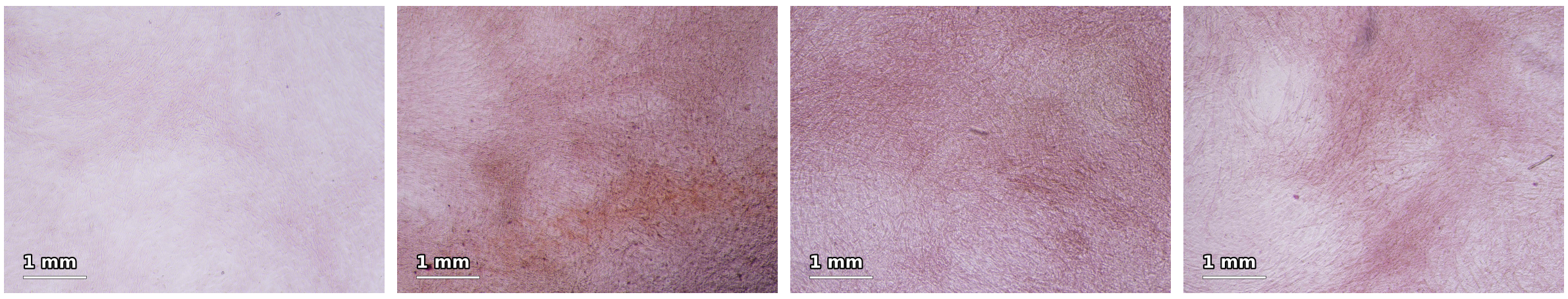

(a) Basal (b) Osteo (c) Osteo + D_L K_L (d) Osteo + D_L K_H

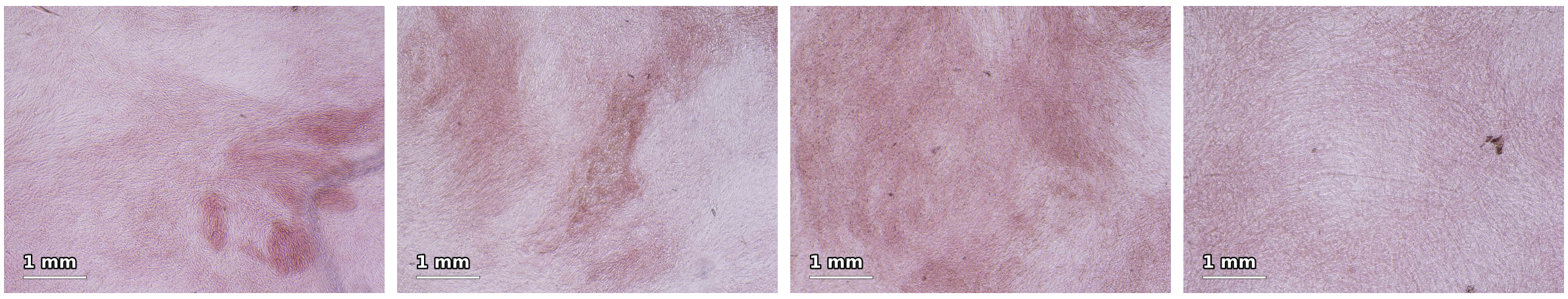

(e) Osteo + D_H K_L (f) Osteo + D_H K_H (g) Osteo + D_H K_0 (h) Osteo + D_0 K_H

Figure 9: Representative Alizarin Red S (ARS) images at day 28 across experimental conditions C1–C8.

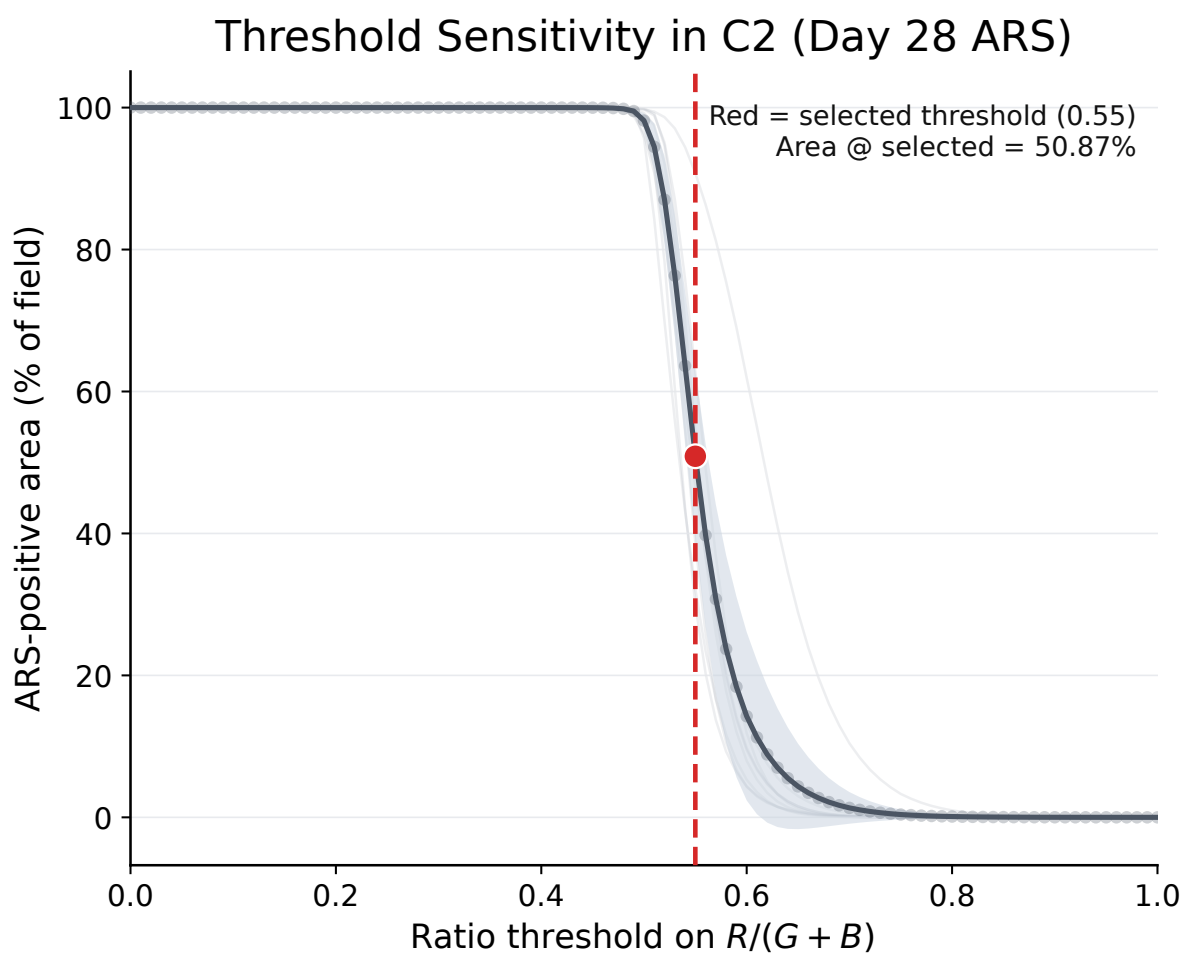

Figure 10: Threshold sensitivity analysis for ARS-positive area quantification in condition C2 at day 28. The dashed red line indicates the selected threshold (0.55), corresponding to an ARS-positive area fraction of 50.87% for the representative image. Shaded curves indicate replicate-level variation.

6 Reagents Information

**Reagent**	Catalog No.	Supplier	Notes
**MC3T3-E1 SubClone 4**	CRL-2593	ATCC	Mouse cell line.
**α-MEM (no ascorbic acid)**	A10490-01	Gibco (Thermo Fisher)	Basal medium.
**Fetal Bovine Serum (FBS)**	F7524-500ML	Sigma-Aldrich	10% v/v supplement.
**Antibiotic–Antimycotic**	A5955-100ML	Sigma-Aldrich	1% v/v supplement.
**DPBS (no Ca, Mg)**	14190136-100ML	Thermo Fisher	Washing buffer.
**Triton X-100**	11498696	Thermo Fisher	Lysis detergent.
**BSA**	A9647	Sigma-Aldrich	Blocking reagent.
**Absolute Ethanol (99.5%)**	733194	Avantor / VWR	Solvent.
**Mowiol 4-88**	17951-1006	Polysciences	Mounting medium.
**Paraformaldehyde (PFA)**	28908	Thermo Fisher	4% fixative.
**TRIS Ultra Pure**	0497-1KG	VWR / Avantor	Buffer base.
**$MgCl_2 \cdot 6H_2O$**	AO344733 230	Merck / Avantor	Analytical grade.
**NaOH**	B1155898 517	Merck / Avantor	pH adjustment.
**L-Ascorbic Acid (AA)**	A4403	Sigma-Aldrich	Supplement.
**β-Glycerophosphate (β-GP)**	G9422	Sigma-Aldrich	Supplement.
**Dexamethasone (Dex)**	D4902	Sigma-Aldrich	Glucocorticoid.
**Vitamin K_2 (MK-4)**	V9378	Sigma-Aldrich	Cofactor.
**Alizarin Red S (ARS)**	A5533	Sigma-Aldrich	Mineral stain.
**Alkaline Phosphatase (pNPP)**	N1891	Sigma-Aldrich	Colorimetric assay.
**Mouse anti-Collagen I**	MA1-26771	Thermo Fisher	Primary antibody.
**Goat anti-Mouse IgG Alexa 488**	A32723	Thermo Fisher	Secondary antibody.
**Phalloidin Alexa 568**	A12380	Thermo Fisher	F-actin stain.
**DAPI**	D1306	Thermo Fisher	Nuclear stain.

References

[1] F. M. Refaaq, X. Chen, and S. W. Pang. Effects of topographical guidance cues on osteoblast cell migration. *Scientific Reports*, 10(1):1–12, 2020. doi: 10.1038/s41598-020-77103-0.

[2] Heein Yoon, Seung Gwa Park, Hye-Rim Shin, Ki-Tae Kim, Young-Dan Cho, Jae-I Moon, Woo-Jin Kim, and Hyun-Mo Ryoo. Unraveling the dynamics of osteoblast differentiation in mc3t3-e1 cells: Transcriptomic insights into matrix mineralization and cell proliferation. *Bone*, 194:117442, 2025. doi: 10.1016/j.bone.2025.117442.

[3] Muhammad Dain Yazid, Shahrul Hisham Zainal Ariffin, Sahidan Senafi, Mohamad Abdul Razak, and Rohaya Megat Abdul Wahab. Determination of the differentiation capacities of murines' primary mononucleated cells and mc3t3-e1 cells. *Cancer Cell International*, 10:42, 2010. doi: 10.1186/1475-2867-10-42.

[4] Dun Hong, Hai-Xiao Chen, Hai-Qiang Yu, Yong Liang, Carrie Wang, Qing-Quan Lian, Hai-Teng Deng, and Ren-Shan Ge. Morphological and proteomic analysis of early stage of osteoblast differentiation in osteoblastic progenitor cells. *Experimental Cell Research*, 316(14):2291–2300, 2010. doi: 10.1016/j.yexcr.2010.05.011.

[5] W. N. Addison, V. Nelea, F. Chicatun, et al. Extracellular matrix mineralization in murine mc3t3-e1 osteoblast cultures. *Bone*, 71:244–256, 2015. doi: 10.1016/j.bone.2014.11.003.

[6] L. M. Luttrell, M. S. Dar, D. Gesty-Palmer, H. M. El-Shewy, et al. Transcriptomic characterization of signaling pathways associated with osteoblastic differentiation of mc3t3-e1 cells. *PLOS ONE*, 14(1):e0204197, 2019. doi: 10.1371/journal.pone.0204197.

[7] Sung-Mi Kim, Yong-Gun Kim, Jin-Woo Park, Jae-Mok Lee, and Jo-Young Suh. The effects of dexamethasone on the apoptosis and osteogenic differentiation of human periodontal ligament cells. *Journal of Periodontal and Implant Science*, 43(4):168–176, 2013. doi: 10.5051/jpis.2013.43.4.168.

[8] Chi-Ming Chiang. Low-dose dexamethasone primes the igf-1 receptor to enhance osteoblast proliferation and mineralization: A mechanistic strategy for hepatic osteodystrophy. *Recent Advances in Clinical Trials*, 5(5):1–7, 2025.

[9] Elena Della Bella, Antoine Buetti-Dinh, Ginevra Licandro, Paras Ahmad, Valentina Basoli, Mauro Alini, and Martin J. Stoddart. Dexamethasone induces changes in osteogenic differentiation of human mesenchymal stromal cells via sox9 and pparg, but not runx2. *International Journal of Molecular Sciences*, 22(9):4785, 2021. doi: 10.3390/ijms22094785.

[10] Weiwei Li, Shaokun Zhang, et al. Vitamin k2 stimulates mc3t3-e1 osteoblast differentiation and mineralization through autophagy induction. *Molecular Medicine Reports*, 19(5):3676–3684, 2019. doi: 10.3892/mmr.2019.10040.

[11] Domitilla Mandatori, Letizia Penolazzi, Caterina Pipino, Pamela Di Tomo, Sara Di Silvestre, Natalia Di Pietro, Sara Trevisani, Marco Angelozzi, Mariangela Ucci, Roberta Piva, and Assunta Pandolfi. Menaquinone-4 enhances osteogenic potential of human amniotic fluid mesenchymal stem cells cultured in 2d and 3d dynamic culture systems. *Journal of Tissue Engineering and Regenerative Medicine*, 12(5):e2490–e2502, 2018. doi: 10.1002/term.2471.

[12] T. Chepda, M. Cadau, Ph. Girin, J. Frey, and A. Chamson. Monitoring of ascorbate at a constant rate in cell culture: Effect on cell growth. *In Vitro Cellular & Developmental Biology - Animal*, 37(1):26–30, 2001. doi: 10.1290/1071-2690(2001)037⟨0026:MOAAAC⟩2.0.CO;2.

[13] N. Fratzl-Zelman, P. Fratzl, H. Hörandner, B. Grabner, F. Varga, A. Ellinger, and K. Klaushofer. Matrix mineralization in mc3t3-e1 cell cultures initiated by beta-glycerophosphate pulse. *Bone*, 23(6):511–520, 1998. doi: 10.1016/S8756-3282(98)00139-2.
